# Mode of progression after radioembolization in patients with colorectal cancer liver metastases

**DOI:** 10.1186/s13550-020-00697-z

**Published:** 2020-09-22

**Authors:** Caren van Roekel, Jennifer M. J. Jongen, Maarten L. J. Smits, Sjoerd G. Elias, Miriam Koopman, Onno Kranenburg, Inne H. M. Borel Rinkes, Marnix G. E. H. Lam

**Affiliations:** 1 Department of Radiology and Nuclear Medicine, University Medical Center Utrecht, University Utrecht, Heidelberglaan 100, 3584 CX Utrecht, The Netherlands; 2 Department of Surgical Oncology, Endocrine and GI Surgery, Cancer Center, University Medical Center Utrecht, University Utrecht, Utrecht, The Netherlands; 3 Julius Center for Health Sciences and Primary Care, University Medical Center Utrecht, University Utrecht, Utrecht, The Netherlands; 4 Department of Medical Oncology, Cancer Center, University Medical Center Utrecht, University Utrecht, Utrecht, The Netherlands; 5 Division of Biomedical Genetics, University Medical Center Utrecht, University Utrecht, Utrecht, The Netherlands

**Keywords:** Metastatic colorectal cancer, Radioembolization, Progression, RECIST, Extrahepatic metastases

## Abstract

**Background:**

Radioembolization is an established treatment modality in colorectal cancer patients with liver-dominant disease in a salvage setting. Selection of patients who will benefit most is of vital importance. The aim of this study was to assess response (and mode of progression) at 3 months after radioembolization and the impact of baseline characteristics.

**Methods:**

Three months after radioembolization with either yttrium-90 resin/glass or holmium-166, anatomic response, according to RECIST 1.1, was evaluated in 90 patients. Correlations between baseline characteristics and efficacy were evaluated. For more detailed analysis of progressive disease as a dismal clinical entity, distinction was made between intra- and extrahepatic progression, and between progression of existing metastases and new metastases.

**Results:**

Forty-two patients (47%) had extrahepatic disease (up to five ≥ 1 cm lung nodules, and ≤ 2 cm lymph nodes) at baseline. No patients showed complete response, 5 (5.5%) patients had partial response, 16 (17.8%) had stable disease, and 69 (76.7%) had progressive disease. Most progressive patients (67/69; 97%) had new metastases (intra-hepatic *N* = 11, extrahepatic *N* = 32; or both *N* = 24). Significantly fewer patients had progressive disease in the group of patients presenting without extrahepatic metastases at baseline (63% versus 93%; *p* = 0.0016). Median overall survival in patients with extrahepatic disease was 6.5 months, versus 10 months in patients without extrahepatic disease at baseline (hazard ratio 1.79, 95%CI 1.24–2.57).

**Conclusions:**

Response at 3-month follow-up and survival were heavily influenced by new metastases. Patients with extrahepatic disease at baseline had a worse outcome compared to patients without.

## Background

Approximately 45% of colorectal cancer patients develop metastases [1, 2]. Without treatment, the median overall survival for colorectal cancer patients with hepatic metastases (mCRC) is only 4.5 months [3]. The liver is the most common site of metastasis: up to 30% of mCRC patients develop hepatic metastases [4, 5]. Radioembolization is a loco-regional treatment option for unresectable, systemic therapy-refractory patients with liver-only or liver-dominant disease [6, 7]. Intra-arterial administration of radioactive microspheres is proven to be safe and effective [8]. Microspheres (approximately 30 μm) are loaded with the radioactive isotope yttrium-90 (^90^Y) or holmium-166 (^166^Ho) and injected through a microcatheter in the hepatic artery [9]. For the treatment of metastatic colorectal cancer, ^90^Y-resin microspheres (SIR-Spheres**®**, Sirtex) are FDA- and CE-approved. ^90^Y -glass microspheres (TheraSphere**®**, BTG/Boston Scientific) and ^166^Ho microspheres (QuiremSpheres**®**, Quirem) are CE-approved for this indication, not FDA-approved. The injected microspheres embolize the microvasculature surrounding the tumor and emit high-energy beta-radiation. The normal liver parenchyma is largely spared since healthy liver tissue is mainly supplied by the portal vein [10–12].

Although assessment of metabolic response has proven added benefit over anatomic response, not being hampered by, i.e., the presence of intra-tumoral necrosis and cystic changes after treatment [13, 14], response of radioembolization in mCRC patients is still mostly evaluated by the Response Evaluation Criteria in Solid Tumors (RECIST) [15–17]. When using these criteria, the results of most clinical studies in metastatic (liver) disease are modest, with many patients experiencing early progressive disease [18–21]. Optimized treatment planning could improve response rates [22, 23], but selecting patients who will benefit most is another vital aspect. An important criterion in patient selection is the definition of liver-dominant disease. The extent of extrahepatic disease we are willing to accept is under constant debate at tumor board meetings in our center, but clear guidance is currently missing, due to the lack of data on this matter. Other prognostic factors that are known to influence response after treatment with radioembolization are (among others) KRAS status, primary tumor location, percentage tumor involvement, and pre-treatment CEA level [19, 24, 25]. These factors could possibly be used in patient selection as well.

The aim of this study was to assess the impact of baseline characteristics on changes in intra- and extrahepatic mCRC disease from baseline to 3 months after radioembolization, across all currently available radioembolization treatment modalities.

## Methods

### Patient selection and study design

A total of 129 chemorefractory, unresectable mCRC patients were treated with radioembolization at our institution between August 2009 and January 2017, predominantly as part of the HEPAR-2 (Holmium  Embolization Particles for Arterial Radiotherapy II) [26], or RADAR trial (RADioembolization: Angiogenic factors and Response) [22]. The studies were conducted in accordance with the institutions’ Medical Ethical Committee and informed consent was obtained from the patients treated in the HEPAR-2 and RADAR studies before inclusion. For the other patients that were treated in routine clinical practice and also included in the current retrospective analysis, the need for informed consent was waived. Inclusion criteria for all patients regarding the presence of extrahepatic metastases or the primary tumor were similar: liver-dominant disease with a maximum of five lung nodules < 1 cm and lymph nodes < 2 cm. The presence of the primary tumor was not a contra-indication to treatment. Patients were included for response analysis in case CT and/or MRI scans were available at baseline and at (around) 3-month follow-up; all patients were included for survival analysis. Patients were treated with ^166^Ho-microspheres (*n* = 24) (all as part of the HEPAR 2 study), glass ^90^Y-microspheres (*n* = 20), or resin ^90^Y-microspheres (*n* = 46). Imaging was performed 3 months after treatment (i.e., whole-liver or lobar treatment in one session). In case of sequential lobar treatment, imaging was performed 3 months after the last lobar treatment.

The electronic medical records were reviewed to obtain patient characteristics. The following established independent prognostic factors in patients with mCRC were compared: age, number of previous chemotherapy lines, type of microspheres, presence of extrahepatic disease at baseline, primary tumor in situ, time since diagnosis of metastases, WHO performance status, KRAS wild type versus KRAS mutation, pre-treatment CEA level, primary tumor location (categorized as left sided (splenic flexure to rectum) or right sided (proximal to the splenic flexure)), and tumor load (percentage liver involvement, categorized as < 25%, 25–50%, > 50%) [21, 25, 27–33].

### Radioembolization

The prescribed activity for the patients that were treated with glass ^90^Y-microspheres was calculated according to the Medical Internal Radiation Dose (MIRD) method, with a desired absorbed dose of 80–120 Gy, according to the instructions for use [34–36]. Visual and quantitative assessment of ^99m^Tc-MAA distribution is weighted in this decision, also considering whole liver treatment in one session or sequentially. For the patients that were treated with resin ^90^Y-microspheres, the body surface area (BSA) method was used. The injected activity for ^166^Ho-microspheres was calculated based on the MIRD method with an aimed whole-liver absorbed dose of 60 Gy [37].

### Response assessments

Two blinded readers independently performed measurements for tumor diameter on abdominal contrast-enhanced CT or MRI at baseline and 3-month follow-up, using the same modality at both time points, according to RECIST version 1.1 [17]. In case no consensus was reached, a third reader gave the final call. Finally, inter-observer variability between the two raters was assessed.

Response at 3 months was dichotomized as disease control (i.e., complete or partial response (CR or PR) and stable disease (SD)) or progressive disease (PD). For a more detailed assessment of mode of progressive disease, a further subdivision was made in four categories: growth of intrahepatic metastases, growth of extrahepatic metastases, new intrahepatic metastases, and new extrahepatic metastases. All extrahepatic metastases were taken into account, regardless of their size.

### Statistical analyses

Standard descriptive statistics were used to display patient demographics and summarize response measures. Cohen’s kappa was used to determine agreement. Chi-square was used to test for differences in whole body response classification. Firth’s logistic regression was used to explore associations between baseline characteristics and mode of progression. This type of analysis was chosen to correct for small-sample bias [38]. The analysis for the association between extrahepatic disease at baseline and disease progression was adjusted for the following possible confounders: time from diagnosis of metastases to treatment, primary tumor in situ, KRAS mutation vs wild type, and number of lines of previous systemic treatment (one versus two or more). The analysis for the association between type of microsphere used and disease progression was adjusted for the following possible confounders: age, time from diagnosis of metastases to treatment, primary tumor in situ, KRAS mutation vs wild type, number of lines of previous systemic treatment (one versus two or more), and presence of extrahepatic disease. Univariable survival analysis by the Kaplan-Meier method was used to estimate median overall survival (OS) in all treated patients. A Cox proportional hazards model with Firth’s correction was used to test for differences in survival between patients with and without extrahepatic disease at baseline. All analyses were performed using R version 3.6.2 for Windows. We report effect estimates with associated 95%CIs and corresponding two-sided *p* values.

## Results

### Patient demographics

Of the total cohort of 129 treated patients in our institution, 39 patients (30%) did not have 3-month follow-up imaging available because of the following reasons: follow-up imaging in other hospitals (*n* = 5), only follow-up imaging at 1 month post-treatment (*n* = 21), only response evaluation using ^18^F-FDG PET (with no accompanying contrast-enhanced CT) (*n* = 5), clinical progression (*n* = 5), no follow-up imaging available (*n* = 2), and RFA artifacts (*n* = 1). The remaining 90 patients had either CT (*n* = 67, 74%) or MRI (*n* = 23, 26%) images available at baseline and 3-month follow-up. Median interval between baseline imaging and radioembolization was 18 days (range 1–46), between radioembolization and follow-up 91 days (range 62–165). Baseline and treatment characteristics are summarized in Table 1. ^166^Ho-microspheres, glass ^90^Y-microspheres, and resin ^90^Y-microspheres were used in 24 (27%), 20 (22%), and 46 patients (51%) respectively. None of the patients received systemic treatment before (< 4 weeks), during, or after (< 3 months) radioembolization.

**Table 1:** Baseline and treatment characteristics

Characteristic	*N* (%) or median with range					
	^*90*^*Y-resin*	^*90*^*Y-glass*	^*166*^*Ho*	*No extrahepatic disease*	*Extrahepatic disease at baseline*	*Total*
*N*	46 (51)	20 (22)	24 (27)	48 (53)	42 (47)	90 (100)
Age (years)	65 (35–84)	67 (45–78)	66 (40–84)	66 (34–84)	66 (40–84)	66 (35–84)
Gender						
Male	33 (72)	15 (75)	17 (63)	34 (71)	31 (74)	65 (72)
Female	13 (28)	5 (25)	7 (37)	14 (29)	11 (26)	25 (28)
WHO performance status						
0	24 (52)	15 (75)	19 (79)	29 (60)	29 (69)	58 (64)
1	19 (41)	5 (25)	5 (21)	18 (38)	11 (26)	29 (32)
2	3 (7)	0 (0)	0 (0)	1 (2)	2 (5)	3 (4)
Previous chemotherapy lines						
0	1 (2)	0 (0)	0 (0)	1 (2)	0 (0)	1 (1)
1	13 (28)	9 (45)	11 (46)	18 (38)	15 (36)	33 (37)
2	21 (46)	8 (40)	11 (46)	21 (44)	9 (21)	40 (44)
3	11 (24)	3 (15)	2 (8)	8 (17)	8 (19)	16 (18)
Bevacizumab	29 (63)	13 (65)	13 (54)	32 (67)	23 (55)	55 (61)
Capecitabine	42 (91)	18 (90)	20 (83)	41 (85)	39 (93)	80 (89)
Cetuximab	2 (4)	0 (0)	1 (4)	1 (2)	2 (5)	3 (4)
Cisplatin	1 (2)	0 (0)	0 (0)	1 (2)	0 (0)	1 (1)
Erlotinib	0 (0)	0 (0)	1 (4)	0 (0)	1 (2)	1 (1)
Irinotecan	26 (57)	9 (45)	10 (42)	23 (48)	22 (52)	45 (50)
Oxaliplatin	40 (87)	17 (85)	23 (96)	41 (85)	39 (93)	80 (89)
Paclitaxel	0 (0)	0 (0)	1 (4)	0 (0)	1 (1)	1 (1)
Panitumumab	8 (17)	4 (20)	3 (13)	9 (19)	6 (14)	15 (17)
5-FU	7 (15)	3 (15)	5 (21)	7 (15)	8 (19)	15 (17)
Previous locoregional treatment						
Yes	17 (37)	7 (35)	6 (25)	17 (35)	13 (31)	30 (33)
No	29 (63)	13 (65)	18 (75)	31 (65)	29 (69)	60 (67)
Metastasis pattern						
Synchronous	31 (67)	15 (75)	15 (63)	27 (56)	34 (81)	61 (68)
Metachronous	15 (33)	5 (25)	9 (37)	21 (44)	8 (19)	29 (32)
Time since diagnosis (months)	25 (3–97)	24 (11–110)	26 (6–92)	26 (3–110)	21 (5–92)	25 (3–110)
Time since diagnosis of metastatic disease (months)	17 (3–72)	23 (2–50)	18 (2–92)	17 (2–54)	21 (5–92)	19 (2–92)
KRAS status						
Wild-type	16 (35)	8 (40)	9 (37)	21 (44)	12 (29)	33 (37)
Mutation	11 (24)	3 (15)	6 (26)	7 (15)	13 (31)	20 (22)
Unknown	19 (41)	9 (45)	9 (37)	10 (21)	17 (40)	37 (41)
CEA level	72 (3–2700)	68 (3–640)	100 (2–6000)	61 (2–2700)	115 (3–6000)	88 (2–6000)
Unknown	12 (26)	4 (20)	1 (4)	7 (15)	10 (24)	17 (19)
Primary tumor in situ						
Yes	1 (2)	4 (20)	2 (8)	2 (4)	5 (12)	7 (8)
No	45 (98)	16 (80)	22 (92)	46 (96)	37 (88)	83 (92)
Extrahepatic disease (all metastases)						
None	27 (59)	9 (45)	10 (42)	48 (100)	0 (0)	48 (53)
Lymph node metastases	12 (26)	8 (40)	7 (29)	-	25 (60)	25 (28)
Lung metastases	8 (17)	2 (10)	7 (29)	-	17 (40)	17 (19)
Abdominal wall metastases	1 (2)	0 (0)	0 (0)	-	1 (2)	1 (1)
Spleen metastases	0 (0)	0 (0)	2 (8)	-	1 (2)	1 (1)
Adrenal gland metastases	0 (0)	2 (10)	0 (0)	-	2 (5)	2 (2)
Peritoneal metastases	1 (2)	0 (0)	2 (8)	-	3 (7)	3 (3)
Type of radioembolization						
Whole-liver	41 (89)	12 (60)	20 (83)	38 (79)	35 (83)	73 (81)
Lobar	5 (11)	8 (40)	4 (17)	10 (21)	7 (17)	17 (19)
Injected activity (MBq)	1526 (636–2320)	2037 (711–6277)	6565 (2213–11,627)	1882 (636–11,164)	1992 (680–11,627)	Not applicable
Lungshunt (%)	5 (0.1–17)	2.3 (1–26)	4.6 (0.3–16)	4.5 (0.1–17)	3.4 (0.8–26)	4 (0.1–26)

This table shows the baseline characteristics for the 90 included patients

### Inter-observer variability

Discordant conclusions were drawn in five patients, for whom the third rater gave the final call. The level of agreement in RECIST categories was adequate with a Cohen’s kappa of 0.895 (95% CI 0.805–0.985), *p* < 0.001.

### Response according to RECIST 1.1

**Fig. 1:**
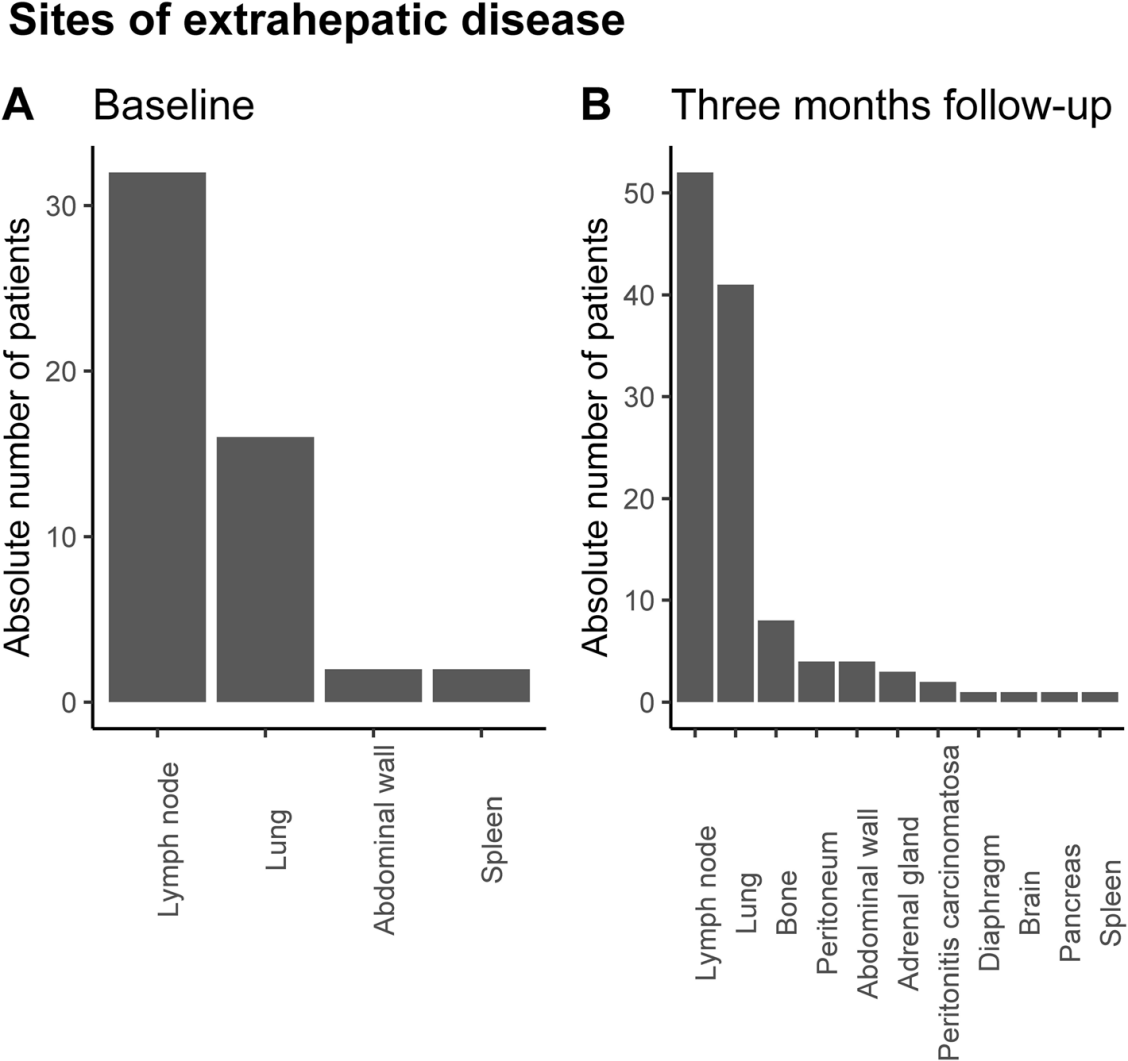
Site of extrahepatic metastases at baseline and at 3-month follow-up. **a** Type and number of affected organs in patients with extrahepatic metastases at baseline. Bars depict absolute number of patients. In total, 50 affected organs in 42 patients. **b** As in **a**, for 3-month follow-up. In total, 119 affected organs in 67 patients. Lymph nodes and lung are most affected at baseline and 3-month follow-up

At baseline, 42/90 (47%) patients had extrahepatic metastases, which increased to 67/90 (74%) patients at 3-month follow-up (Fig. 1).

**Fig. 2:**
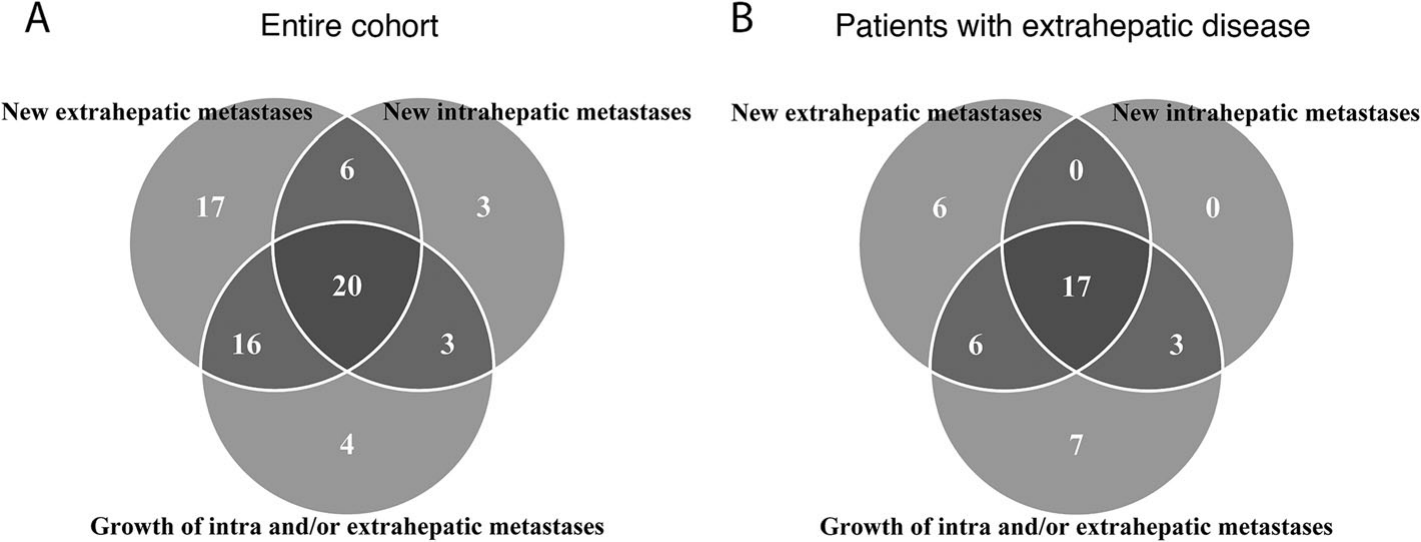
Mode of progression of the entire cohort (90 patients) (**a**) and specifically for patients with extrahepatic disease at baseline (42 patients) (**b**). Numbers indicate the number of patients in the indicated (overlapping) group

Of the 90 patients, no patients showed CR, 5 (5.5%) patients had PR, 16 (17.8%) had SD, and 69 (76.7%) had PD. According to RECIST, progressive disease can be based on growth of intrahepatic metastases, growth of extrahepatic metastases, or new metastases (either intra- or extrahepatic). Growth of intrahepatic metastases was observed in 20 patients (29%), new intrahepatic metastases in 35 patients (51%), growth of extrahepatic metastases in 37 patients (54%), and 56 patients (81%) were diagnosed with new extrahepatic metastases. Most, 67/69 of the progressive patients (97%), had new (intra-hepatic *N* = 11, extrahepatic *N* = 32; or both *N* = 24) metastases. Progression was most often seen on multiple levels (*N* = 42, 61%) and was only based on growth of existing metastases in 5 patients (7%, intra-hepatic *N* = 2, extrahepatic *N* = 3) and on only new lesions in 23 patients (69%) (Fig. 2a). In the subgroup of progressive patients with extrahepatic disease at baseline, new extrahepatic metastases were most common, in 28/42 (67%) patients (Fig. 2b).

There was no significant difference in response between the three types of microspheres used: compared to ^90^Y resin microspheres, the odds ratios for progressive disease with ^90^Y glass and ^166^Ho were 1.11 (95%CI 0.32–4.53) and 0.67 (95%CI 0.22–2.14), respectively (Table 2).

**Table 2:** Univariable Firth’s logistic regression analysis for baseline characteristics and type of progression: OR (95%CI)

	PD	GIHM (*n* = 20)	GEHM (*n* = 37)	NIHM (*n* = 35)	NEHM (*n* = 57)	GM (*n* = 45)	NM (*n* = 63)
Age in years	1.02 (0.97; 1.07)	0.99 (0.02; 9.75)	1.01 (0.97; 1.06)	1.02 (0.98; 1.06)	1.00 (0.96; 1.05)	1.01 (0.97; 1.06)	1.01 (0.97; 1.05)
Chemotherapy*	0.88 (0.01; 88.46)	0.74 (0.26; 2.01)	0.87 (0.37; 2.05)	0.37 (0.14; 0.91)	0.63 (0.26; 1.51)	0.57 (0.24; 1.33)	0.48 (0.19; 1.19)
Type of microspheres**, Glass	1.11 (0.32; 4.53)	0.71 (0.18; 2.41)	2.53 (0.87; 7.56)	1.71 (0.59; 4.99)	1.5 (0.50; 4.89)	1.79 (0.62; 5.35)	1.94 (0.59–7.65)
Type of microspheres, holmium	0.67 (0.22; 2.14)	0.57 (0.14; 1.88)	1.75 (0.63; 4.86)	0.85 (0.29; 2.38)	0.90 (0.33; 2.50)	1.19 (0.44; 3.22)	0.81 (0.29–2.31)
Extrahepatic metastases at baseline	*7.8 (2.37; 35.53)*	2.39 (0.89; 6.84)	NA	1.83 (0.79; 4.32)	*3.06 (1.28; 7.72)*	NA	*2.86 (1.14; 7.66)*
Primary tumor in situ	1.90 (0.30; 37.11)	0.76 (0.08; 3.96)	1.12 (0.24; 4.88)	3.86 (0.87; 22.5)	2.73 (0.54; 27.1)	1.32 (0.30; 6.21)	2.00 (0.39; 19.9)
WHO status***	0.87 (0.32; 2.46)	0.43 (0.10; 1.43)	0.91 (0.35; 2.33)	0.90 (0.33; 2.33)	0.50 (0.19; 1.31)	0.86 (0.34; 2.19)	0.81 (0.30; 2.27)
KRAS status^§^	3.38 (0.75; 23.95)	1.88 (0.56; 6.45)	2.75 (0.90; 8.78)	4.78 (1.52; 16.5)	3.92 (1.11; 17.2)	2.85 (0.92; 9.48)	2.67 (0.74; 11.86)
Time since diagnosis of metastases^§§^	*1.06 (1.01; 1.11)*	0.98 (0.94; 1.01)	1.01 (0.99; 1.04)	0.98 (0.95; 1.01)	1.02 (0.99; 1.05)	1.01 (0.98; 1.03)	1.02 (0.99; 1.07)
CEA level before treatment	1.00 (0.99–1.00)	1.00 (95%CI 0.99–1.00)	1.00 (95%CI 0.99–1.00)	1.00 (95%CI 0.99–1.00)	1.00 (0.99–1.00)	1.00 (0.99–1.01)	1.00 (0.99–1.01)
Primary tumor location	3.88 (1.00–25.75)	0.88 (0.26–2.63)	0.98 (0.35–2.63)	1.72 (0.65–4.59)	2.96 (0.98–11.11)	0.94 (0.36–2.49)	3.88 (1.00–25.76)
Tumor load^†^, 25–50%	1.63 (0.50–6.35)	0.56 (0.14–1.79)	1.17 (0.42–3.16)	0.58 (0.20–1.59)	0.94 (0.34–2.72)	1.04 (0.39–2.74)	1.11 (0.36–3.90), 0.7
Tumor load, > 50%	0.73 (0.20–3.10)	1.24 (0.30–4.49)	1.67 (0.47–5.76)	1.64 (0.48–5.76)	0.55 (0.16–1.95)	1.66 (0.49–6.12)	0.66 (0.18–2.77)

This table shows the associations (odds ratio and 95%CI) between baseline characteristics and modes of progression as described by RECIST at 3 months post-treatment

*Abbreviations: GIHM* growth of intrahepatic metastases, *GEHM* growth of extrahepatic metastases, *NIHM* new intrahepatic metastases, *NEHM* new extrahepatic metastases, *GM* growth of metastases (GEHM + GIHM), *NM* new metastases (NEHM + NIHM), *NA* not applicable, *RE* radioembolization, *OR* odds ratio, *PD* progressive disease, *95% CI* 95% confidence interval

*Previous treatment with 1st- versus 2nd-line chemotherapy

**Yttrium-90 resin (reference) versus yttrium-90 glass and holmium-166

***WHO performance status 0 versus 1, 2

^§^KRAS mutation versus KRAS wild type

^§§^Right-sided primary tumors versus left-sided primary tumors

^†^< 25% liver involvement versus 25–50% and > 50% liver involvement

### Correlations between baseline characteristics and response

**Fig. 3:**
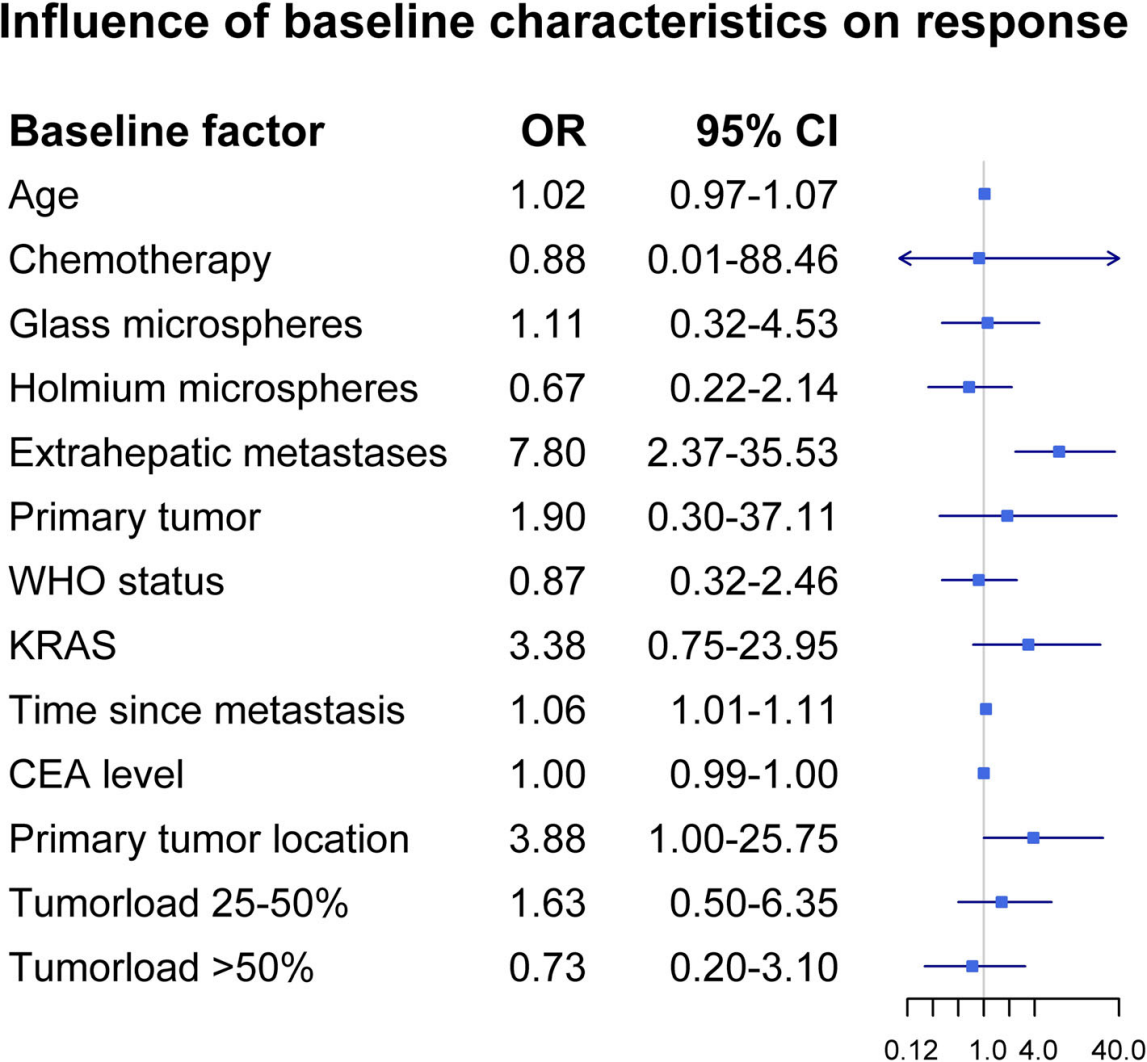
Forest plot of the influence of baseline characteristics on response (progression versus no progression according to RECIST 1.1), based on univariable analyses

The association between several baseline characteristics and response was assessed (Table 2). Presence of extrahepatic disease was the most significant risk factor for progressive disease, with an OR of 7.8 (95% CI 2.37–35.53) for patients with extrahepatic disease at baseline versus patients without extrahepatic disease at baseline (Fig. 3). Extrahepatic metastases at baseline increased the risk of progressive disease for all modes of progression, mainly for new extrahepatic metastases (OR = 3.06, 95% CI 1.28–7.72). Time since diagnosis of metastases was a significant risk factor for progressive disease as well, with an OR of 1.06 for every month increase in time (95%CI 1.01–1.11). Primary tumor location showed a strong trend, with an OR of 3.88 (95%CI 1.00–25.75) for patients with right-sided primary tumors versus patients with left-sided primary tumors. There was no significant difference between types of microspheres used.

The difference in response evaluation was compared for patients with or without extrahepatic metastases at baseline. Of the group (*n* = 42, 47%) presenting with extrahepatic metastases at baseline, 93% was diagnosed with PD at 3-month follow-up. Significantly fewer patients (63%) were diagnosed with progressive disease in the group of patients (*n* = 48, 53%) presenting without extrahepatic metastases at baseline (*p* = 0.0017) (Table 3).

**Table 3:** RECIST 1.1 response classification at 3 months post-treatment

Total	No extrahepatic metastases at baseline		48(53%)
		Complete response Partial response Stable disease Progressive disease	0 (0%) 5 (10%) 13 (27%) 30 (63%)
	Extrahepatic metastases at baseline		42 (47%)
		Complete response Partial response Stable disease Progressive disease	0 (0%) 0 (0%) 3 (7%) 39 (93%)*
Yttrium-90 Resin	No extrahepatic metastases at baseline		28(61%)
		Complete response Partial response Stable disease Progressive disease	0 (0%) 1 (4%) 7 (25%) 20 (71%)
	Extrahepatic metastases at baseline		18 (39%)
		Complete response Partial response Stable disease Progressive disease	0 (0%) 0 (0%) 2 (11%) 16 (89%)
Yttrium-90 Glass	No extrahepatic metastases at baseline		9 (45%)
		Complete response Partial response Stable disease Progressive disease	0 (0%) 2 (22%) 2 (22%) 5 (56%)
	Extrahepatic metastases at baseline		11 (55%)
		Complete response Partial response Stable disease Progressive disease	0 (0%) 0 (0%) 0 (0%) 11 (100%)
Holmium-166	No extrahepatic metastases at baseline		11 (46%)
		Complete response Partial response Stable disease Progressive disease	0 (0%) 2 (18%) 4 (36%) 5 (46%)
	Extrahepatic metastases at baseline		13 (54%)
		Complete response Partial response Stable disease Progressive disease	0 (0%) 0 (0%) 1 (8%) 12 (92%) *

This table shows a comparison of RECIST 1.1 response classification at 3 months post-treatment for patients with or without extrahepatic metastases at baseline. Numbers represent number of patients (% of total/subcategory)

*Marks significant difference between groups; *p* < 0.05

### Prognostic value of extrahepatic disease at baseline based on overall survival (OS)

**Fig. 4:**
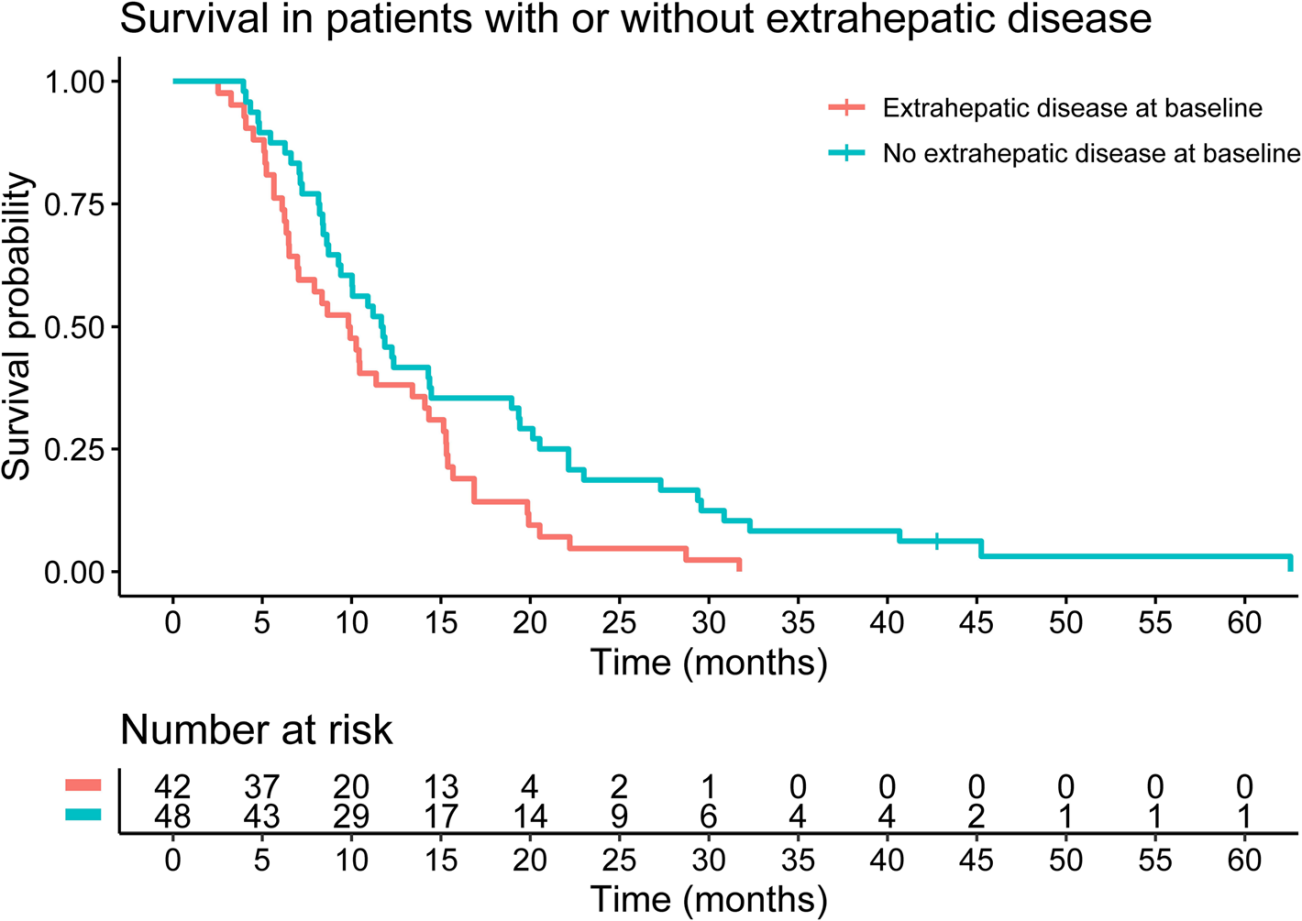
Kaplan-Meier survival curve stratified for extrahepatic metastases at baseline

Median OS for the 90 included patients was 10 months (95% CI 9–14 months). Presence of extrahepatic metastases at baseline showed a difference in median OS estimates with 10 months (95% CI 7–14) for patients with and 12 months (95% CI 9–19) for patients without extrahepatic metastases at baseline (hazard ratio (HR) 1.68, 95%CI (1.09–2.59), *p* = 0.019) (Fig. 4).

## Discussion

This study shows that a large proportion of end-stage mCRC patients have progressive disease after radioembolization due to the development of new metastases, and to a lesser extent due to the growth of existing metastases. The presence of extrahepatic disease at baseline significantly increases the chance of early progressive disease at 3 months, especially the development of new metastases. Moreover, patients with extrahepatic metastases at baseline had a significantly worse overall survival.

At baseline, 48% of our study population was diagnosed with extrahepatic metastases. This is in line with other studies in which 35–77% of the included patients had extrahepatic metastases at baseline [18–20, 39–44]. We found a difference in median OS with and without the presence of extrahepatic metastases at baseline, respectively 7 versus 10 months (*p* = 0.0018). Several other studies with a comparable patient population also found that extrahepatic disease was a predictor of survival after radioembolization [24, 45–49]. Other known prognostic factors are tumor load, baseline CEA level, and location (left- versus right-sidedness) of the primary tumor [24, 49, 50]. In our study, only location of the primary tumor showed a clear trend for significance, with the odds ratio for progressive disease being 3.88 (95%CI 1.00–25.75) for patients with a right-sided primary tumor versus patients with a left-sided primary tumor.

Genetics and biomarkers are more and more recognized as prognostic factors. We investigated the possible role of CEA, since this was associated with poorer survival after radioembolization in multiple studies [19, 24, 51]. However, just as in the study of Sofocleus et al., in our study, no significant correlation between pre-treatment CEA level and disease progression was found [19]. Patients with KRAS mutation generally have a worse prognosis after radioembolization than patients with KRAS wild-type status [19, 24, 25, 52]. In our study, although not significant, the odds ratios for all types of progressive disease showed a clear trend for a worse prognosis for patients with KRAS mutation versus patients with KRAS wild type (Table 2).

In The Netherlands, indications for radioembolization include liver-dominant, irresectable, systemic therapy-refractory disease. Patients with significant extrahepatic metastases are not considered eligible, but patients with stable, limited extrahepatic disease (defined by the Dutch National Healthcare Institute as a maximum of 5 lung nodules < 1 cm and lymph nodes < 2 cm) are eligible [53]. This criterion was also used in the patients in this study. The SIRFLOX, FOXFIRE, and FOXFIRE-Global (studying the added value of radioembolization to chemotherapy in first-line mCRC patients) used similar inclusion criteria with respect to extrahepatic disease [54]. In these studies, no difference in OS or overall progression-free survival (PFS) was observed [55]. One may argue that the large percentage of patients with extrahepatic disease in these studies (i.e., 36%) clouded the potential clinical benefit of radioembolization in a more stringent selected subset. In a subgroup of patients with right-sided primary tumors, the presence of extrahepatic metastases at baseline indeed proved to be a negative prognostic factor for OS, with a HR of 1.351 (95%CI 0.96–1.91) [50]. Importantly, these studies were performed in first-line refractory disease. This limits comparison with our study in a more advanced-stage population.

Objective response (CR or PR) at 3 months after treatment was obtained in only 6% of our patients. This is in line with other studies in salvage mCRC patients, with reported response ranges of 6–24% [24, 56, 57]. Median OS in our study was 10 months, which is also in line with other studies in a comparable patient population [22, 24, 58].

A reason for the modest treatment results in our study might be the dosimetric models that were used: the BSA and MIRD methods. These methods can lead to underdosing [59, 60]. A personalized treatment approach, as was used in the DOSISPHERE study in HCC patients, could have led to a much higher response rate [61]. The results of earlier studies on the dose-response relations in mCRC patients treated with ^90^Y-resin or ^166^Ho prove this point: a significant dose-response relationship was found in both studies [22, 62]. Implementing the results of these studies in future patients, using an individualized treatment approach, likely will lead to a higher treatment accuracy.

In our study, response was evaluated using the anatomic criteria as defined by the RECIST guidelines. However, this can be hampered by the presence of necrosis, hemorrhage, and cystic changes [63]. Response assessment based on changes in functional metrics as determined on [^18^F]-FDG PET/CT would be a better evaluation method, especially since several studies found that these are related with overall survival [13, 22, 64, 65]. Unfortunately, not all patients in our study underwent baseline and post-treatment imaging by [^18^F]-FDG PET/CT.

The added value of the present study to the existing knowledge on radioembolization in mCRC patients is the fact that the development of new metastases is the primary cause for progressive disease after treatment. Furthermore, the study shows that the development of new lesions, as well as progressive disease in general, is more common in patients with extrahepatic disease at baseline.

The current study also has several limitations. First of all, the sample size was small. Secondly, the retrospective setting was prone to selection bias. Since radioembolization was used in a salvage setting, outcome was likely muddled by the effect of other, previous therapies (Table 1). However, since patients were selected for radioembolization based on their chemo-resistant tumors, the contribution of this variation in our patient population on the outcome of our study was considered minimal. Third, all patients were discussed in a multidisciplinary tumor board before treatment. Based on available imaging, the primary tumor was assessed for stability and the extrahepatic disease load was assessed for extent, however, not for stability. Also, although radioembolization is nowadays often performed in a lobar approach, a large fraction of patients that we studied received whole-liver treatment. Whole-liver treatment was in large part dictated by study protocols. Furthermore, three types of microspheres were used in our dataset. The differences with regard to the embolic nature of the treatment, the specific activity of the microspheres, the administered activities, and the absorbed doses may have influenced the incidence of early progressive disease, and potentially also the mode of progression, although our analyses did not show a significant difference between microsphere types. Last, KRAS status was unknown in 42% of the patients, making the number of patients for the subgroup analyses for KRAS rather small.

Proper selection of patients seems fundamental for the cost-effectiveness of radioembolization treatment. Future prospective studies in the salvage setting should therefore be conservative with regard to the acceptance of extrahepatic disease. Accurate baseline imaging, including FDG-PET, may aid patient selection [66]. This will avoid futile treatments and unnecessary toxicity. However, the effect of radioembolization in patients with extrahepatic disease should be evaluated in prospective studies comparing radioembolization with best supportive care, before a firm statement can be made about the exclusion of patients with extrahepatic disease from treatment. Also, considering the development of new lesions as the major cause of progressive disease, a study in the third line, comparing TAS-102 or regorafenib with and without radioembolization, would be interesting. The study of Hendlisz et al. showed that radioembolization combined with chemotherapy was safe and effective [58]. Based on the results of this study, chemotherapy in addition to radioembolization was therefore recommended in the refractory setting.

Proper selection and individualized dosimetry-based treatment planning should ultimately lead to improved treatment accuracy in mCRC patients.

## Conclusions

In conclusion, response at 3-month follow-up and survival were heavily influenced by new intra- and extrahepatic metastases. Patients with extrahepatic disease at baseline had a worse outcome compared to patients without extrahepatic disease at baseline. Based on the results of this observational, retrospective study, extrahepatic disease may be considered a contraindication for treatment with radioembolization.

## Data Availability

The datasets used and/or analyzed during the current study are available from the corresponding author on reasonable request.

## Abbreviations

^90^Y: Yttrium-90
^166^Ho: Holmium-166
BSA: Body surface area
CR: Complete response
CRC: Colorectal cancer
mCRC: Colorectal cancer metastases
MIRD: Medical Internal Radiation Dose
OS: Overall survival
HCC: Hepatocellular carcinoma
HEPAR-2: Holmium Embolization Particles for Arterial Radiotherapy II
HR: Hazard ratio
PD: Progressive disease
PFS: Progression-free survival
PR: Partial response
RADAR: RADioembolization: Angiogenic factors and Response
RECIST: Response evaluation criteria in solid tumors
SD: Stable disease
